# Lignin as a Partial Polyol Replacement in Polyurethane Flexible Foam

**DOI:** 10.3390/molecules26082302

**Published:** 2021-04-15

**Authors:** Akash Gondaliya, Mojgan Nejad

**Affiliations:** 1 Chemical Engineering and Materials Science, Michigan State University, East Lansing, MI 48824, USA; gondaliy@msu.edu; 2 Department of Forestry, Michigan State University, East Lansing, MI 48824, USA

**Keywords:** lignin, polyurethane (PU), flexible foam, biobased

## Abstract

This study was focused on evaluating the suitability of a wide range of lignins, a natural polymer isolated from different plant sources and chemical extractions, in replacing 20 wt.% of petroleum-based polyol in the formulation of PU flexible foams. The main goal was to investigate the effect of unmodified lignin incorporation on the foam’s structural, mechanical, and thermal properties. The hydroxyl contents of the commercial lignins were measured using phosphorus nuclear magnetic resonance (^31^P NMR) spectroscopy, molar mass distributions with gel permeation chromatography (GPC), and thermal properties with differential scanning calorimetry (DSC) techniques. The results showed that incorporating 20 wt.% lignin increased tensile, compression, tear propagation strengths, thermal stability, and the support factor of the developed PU flexible foams. Additionally, statistical analysis of the results showed that foam properties such as density and compression force deflection were positively correlated with lignin’s total hydroxyl content. Studying correlations between lignin properties and the performance of the developed lignin-based PU foams showed that lignins with low hydroxyl content, high flexibility (low T_g_), and high solubility in the co-polyol are better candidates for partially substituting petroleum-based polyols in the formulation of flexible PU foams intended for the automotive applications.

## 1. Introduction

Polyurethanes (PUs) are defined as polymers containing urethane linkages (–NH–(C=O)–O–), which are formed during the polyaddition reaction of a polyol with di- or poly-isocyanate (–N=C=O)-containing compounds [1]. PUs are versatile polymers that can be manufactured in a wide range of densities, morphologies, and stiffnesses, facilitating their applications in coatings, adhesives, foams, elastomers, and fibers [2]. PU foams accounted for the largest market share of all PU products and are categorized as rigid, semi-rigid, or flexible foams, depending on their core densities, stiffness, and mechanical performances [2]. According to the global forecast reports, the PU foams market was valued at USD 50.2 billion in 2017 and is projected to reach USD 79.8 billion by 2023, growing at an 8% CAGR during 2017–2023 [3]. Polyol is one of the main components of polyurethane foams, with weight fractions ranging from 30–70 wt.% depending on the foams’ final application [2]. Currently, the majority of PU foams are manufactured using fossil-fuel-based polyols. The advancement of society’s interests toward more sustainable products and technologies has motivated both researchers and industries to look for renewable feedstocks [4,5,6].

Several renewable raw materials have been used as polyol replacement in the synthesis of PU foams, such as palm oil [7,8], rapeseed oil [9], sunflower oil [10], soybean oil [11], and castor oil [12]. However, vegetable oils have unsaturated double bonds, which need to be converted to hydroxyl groups through a series of initial steps such as epoxidation, hydroformylation, and reduction/ozonolysis, which could potentially increase the production cost and time [13]. Other biobased polyols derived from industrial byproducts such as wood-based polyols [14,15], and bio-pitch (generated during eucalyptus charcoal production) [16] have also been studied for the production of sustainable and eco-friendly polyurethane flexible foams. The foams produced using bio-pitch were reported to have very high density in the range of 100–160 kg/m^3^ and had low thermal stability compared to petroleum-based flexible PU foams [16].

Among naturally occurring raw materials, lignin is a natural polyol and a promising candidate for producing biobased polyurethanes. Lignin is one of the main components of the plant cell walls, and thus is abundantly available in nature. Lignin can contain up to 15–35 wt.% of the plant’s mass on a dry basis [17]. In 2017, it was reported that nearly 140 million tons of lignin was generated just by pulp and paper industries as byproduct [18]. Most of these lignins are used as a source of fuel to generate power for the papermaking machine, resulting in using lignin at its lowest value [19,20]. Lignin is comprised of complex chemical structures with aliphatic and phenolic hydroxyl groups (similar to hydroxyl groups in petroleum-based polyols), which can react with isocyanate to form urethane linkages [21,22,23]. Several studies have shown that lignin incorporation not only increases the biobased content of the polyurethane products but also provides better performance advantages, such as improving the UV-stability [24], mechanical strength [19], fire retardancy [25,26], biodegradability [27], and antioxidant [24] properties of the final PU foams.

There are different types of lignins whose characteristic structures and chemical properties depend on the delignification process and type of biomass [28]. Lignin samples extracted from biorefining processes and various chemical pulping processes such as kraft [4,19,29,30,31], soda [5,6], and sulfite [32] have been studied to replace polyol in polyurethane flexible foam synthesis.

There are specific challenges in employing lignin, particularly in the PU flexible foam formulations, such as relatively high hydroxyl functional groups of lignin, which increase the crosslinking density, hence producing foam with rigid nature [33]. Other barriers to the utilization of lignin in the development of flexible PU foam are the high glass transition (T_g_) temperature of lignin, which typically ranges between 90–180 °C [34,35,36,37,38], low solubility in the co-polyol [39], and unpleasant odor due to sulfur, which is mainly noticeable in some kraft softwood lignins [39].

Very few studies incorporated lignin in the production of flexible PU foam compared to rigid foams. The previous work indicated that the lignin could be used after chemical modification (of lignin) into PU flexible foams via different methods such as oxypropylation [5], chemical grafting [19], heat treatment [31], or after microwave liquefication [4,5,6]. Bernardini et al. [5] reported that the maximum replacement of polyol with oxypropylated lignin after microwave pretreatment was 25.8 wt.%, and the produced lignin-based PU foam exhibited better thermo-mechanical characteristics. Wang et al. [19] grafted long polyethylene glycol chains (PEG 2000) onto the kraft lignin to improve lignin-based PU foam’s flexibility. They reported that the developed foams were highly resilient even after replacing 50 wt.% of the polyol with PEG grafted lignin. Jeong et al. [31] used unmodified kraft lignin to replace 30 wt.% of the total polyol by dissolving lignin into the co-polyol at a higher temperature (at 60 °C). A study performed by Carriço et al. [29], which used unmodified eucalyptus kraft lignin, resulted in semi-rigid foams with lignin loading of 17.5 wt.%. They showed that developed foams had homogeneous cell sizes with improved thermal and dimensional stability. Lignosulfonate lignin has also been used to synthesize flexible foams [32], and it was observed that the foams developed with lignosulfonate lignin (20–30 wt.% of the total polyol) had higher glass transition temperatures (36–100 °C) than foam made with kraft lignins [32].

Most of these studies focused on using either chemically or thermally modified lignins that require energy, such as heat, microwave treatment to homogenize the phases, or additional chemicals to prepare liquid lignin-polyol (oxypropylation, grafting, or hydrolysis). Using lignin (as it is) without any modification or pretreatment can improve the economic aspects of these renewable raw materials in substituting petrochemicals without using extra energy or chemicals [5]. To the best of our knowledge, the maximum amount of unmodified lignin incorporated in flexible PU foam synthesis was around 17.5 wt.% derived from the kraft hardwood (eucalyptus) pulp [29].

To increase lignin incorporation, it is crucial to understand the effect of lignin’s intrinsic properties extracted from various chemical processes and different biomass sources on the performance of developed lignin-based PU foams. This project was focused on comparing the effects of different lignin sources, extraction processes, and properties on the structural, mechanical, and thermal properties of the developed PU flexible foams. Fifteen different unmodified lignin samples derived from kraft, sulfite, and organosolv processes and various plant sources (softwood, hardwood, and agricultural residues) were used to formulate flexible PU foams using ‘one-pot’ synthesis by replacing 20 wt.% of the petroleum-based polyol. The developed foam’s performance was measured and compared to the performance of a control (reference) foam formulated in the lab without lignin, and with Original Equipment Manufacturer (OEM) standard requirements (automotive applications).

## 2. Results and Discussions

Table 1 shows lignin characterization results, including hydroxyl content, molecular weight distribution, and glass transition temperature (T_g_) of the commercial lignins acquired from various sources and processes.

### 2.1. Lignin Characterization

The total hydroxyl content of lignin samples ranged between 3.48–6.79 mmol/g, depending on the extraction process and the biomass source. These values are significantly higher than the OH-content of the polyether-polyol (0.50 mmol/g) commonly used for flexible foam formulations. The reported hydroxyl content is based on the total hydroxyl content (OH) of the lignin, which comprises aliphatic, phenolic, and carboxylic hydroxyl groups. It was observed that the average hydroxyl content of lignins extracted from the organosolv processes (4.26 mmol/g) were lower than the average hydroxyl content of lignins obtained from the kraft processes (5.58 mmol/g).

The glass transition temperatures of the unmodified lignins were in the range of 84–166 °C. Similarly, it was observed that organosolv lignins had lower T_g_ (84–143 °C) and thus higher flexibility compared to most kraft lignin samples (134–166 °C), as also reported by previous studies [32]. Molecular weight (Mw) data of lignin samples in Table 1 shows that number average molecular weights (Mn) were between 1030–2250 Da; the weight average molecular weights (Mw) of lignin were in the range of 1740–12100 Da, whereas the molecular weight of the polyether polyol used in this study was 6000 Da.

### 2.2. Apparent Density

**Figure 1:**
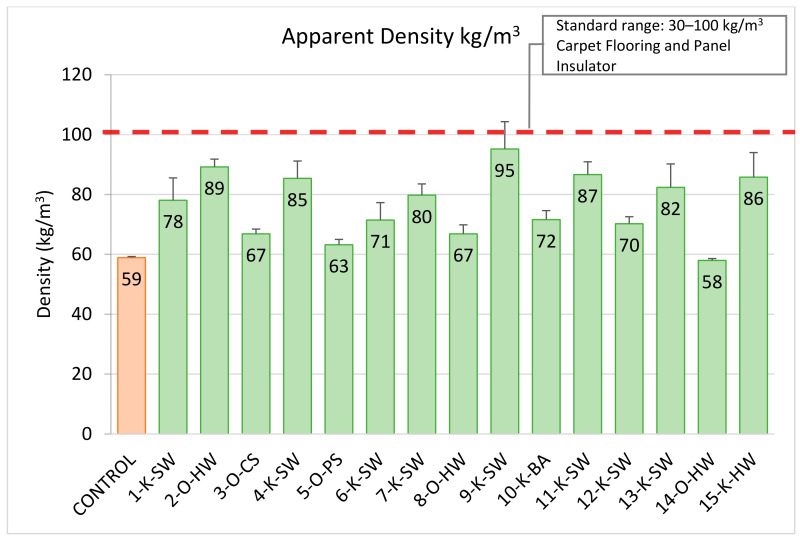
Apparent density of lignin-based PU flexible foams (replacing 20 wt.% of polyol) and control foam (without lignin). **Note:** Orange bar represents the control foam without lignin. Green bars indicate the foam samples that meet the standard requirement.

**Figure 2:**
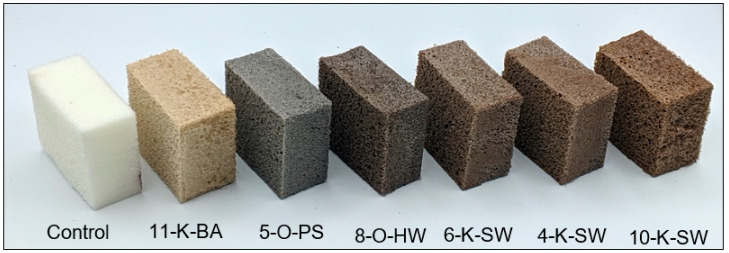
Photos of some of the lignin-based flexible PU foams and control (without lignin, left).

PU flexible foams are widely used in the seating cushions, headrests, and carpet floorings of the cars due to their low density and high performance, improving fuel efficiency in automotive applications. The apparent density (Figure 1) of lignin-containing PU flexible foams was in the range of 58–95 kg/m^3^ and for the control (without lignin) it was 59 kg/m^3^. Figure 2 shows a few of the lignin-based PU flexible foams and the control foam samples used for the density measurements. All lignin-based foams had densities in the standard acceptable range for automotive applications, especially for the panel insulator or floor carpet, which is between 30–100 kg/m^3^ [40,41]. Foams made with organosolv lignins had an average density (69 kg/m^3^), which is significantly lower than the average density of the foams made with the kraft process (81 kg/m^3^). This is potentially due to the fact that organosolv lignins had lower OH content (Ave. 4.24 mmol/g) than kraft lignins (Ave. 5.59 mmol/g), which result in a higher crosslinking density of kraft foams.

### 2.3. Compression Force Deflection (CFD) and Compression Modulus

**Figure 3:**
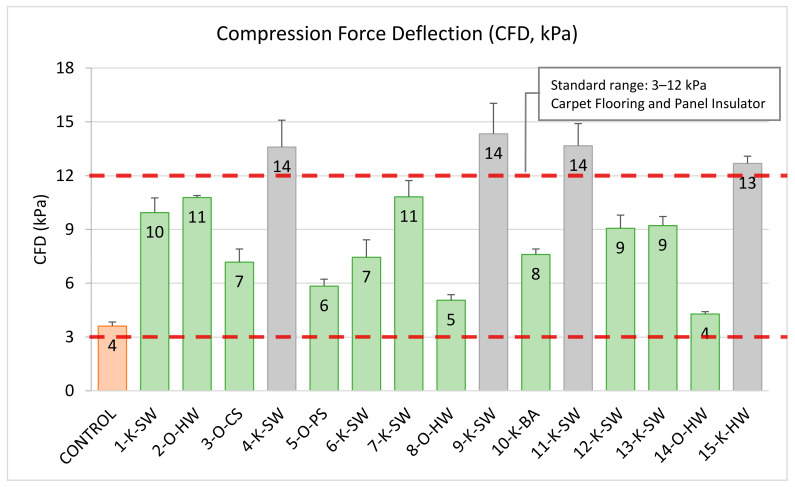
Compression Force Deflection (CFD) test results (ASTM D 3574) of 20 wt.% lignin-based foams and control. **Note**: Orange bar represents the control foam without lignin. Green Bars indicate the foams that meet the standard requirement, and grey bars do not meet the standard automotive requirement for the carpet flooring and panel insulator application.

Compressive force-deflection (CFD) measures the force needed to produce a 50% compression over the foam specimen’s entire upper area. CFD values of lignin-based PU foams for 50% compression are shown in Figure 3. The compression moduli (Table 2) and the CFD values (Figure 3) of the PU foams increased significantly when 20 wt.% of polyol was replaced with lignin, which is in agreement with the previous studies performed on determining the effect of lignin on the mechanical properties of flexible foam [5,6,29]. The main reason for the change in mechanical properties, especially CFD, is due to the higher hydroxyl content (Table 1) of lignins (3.48–6.79 mmol/g) compared to petroleum-based polyol (0.50 mmol/g), resulting in foams with potentially higher amounts of urethane linkages, thus strengthening the struts of lignin-containing PU flexible foams [19].

Moreover, lignin is an aromatic polyol with T_g_ that ranges between 84–166 °C (Table 1); therefore, partially replacing the soft segment of PU flexible foam (polyol) with lignin will reduce the foam’s flexibility and increase its mechanical strengths. Previous studies reported that lignin could act both as a filler and as a crosslinking agent, especially at high incorporation in the PU flexible foams [19,31]. This is mainly evident when the solid lignin has poor solubility in co-polyol. We assume lignins with higher CFD values probably had lower solubility in the co-polyol. Further study is required to verify this hypothesis; it is possible that at higher lignin incorporation, the potential filler effect of lignin will be more evident.

In general, all the developed lignin-based foams can be used in different PU foam applications depending on the CFD values. For instance, foams with lower CFDs are more suitable for car seating applications, while foams with higher CFD are suitable for heavy-duty construction and other parts of automotive. The standard required CFD range for the floor carpet, and panel insulator application in the automotive industry is 3–12 kPa [40]. As shown in Figure 3, all organosolv lignin-based foams had CFD values in this range (green bars). The grey color bars (Figure 3) represent the foam samples that exceeded the carpet flooring and panel insulator application’s maximum limit. This is potentially due to the higher hydroxyl content of the kraft lignins used in the formulation, which led to an increase in the crosslinking density and mechanical strength.

### 2.4. Tear Strength

**Figure 4:**
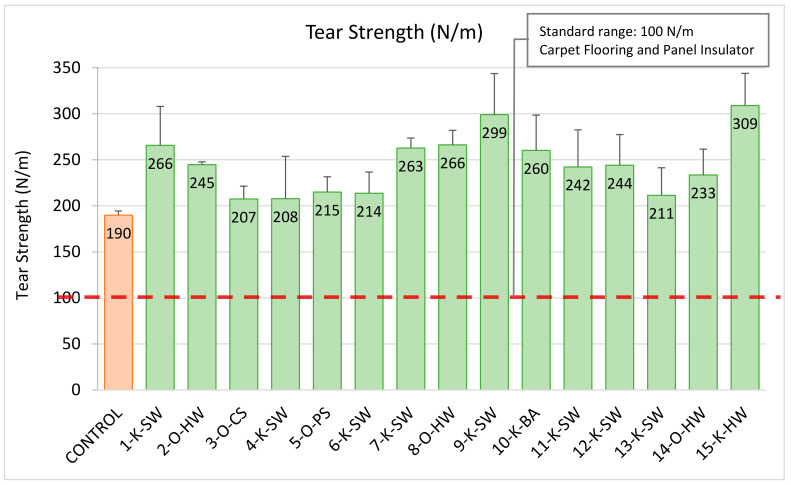
Tear strength (ASTM D 3574) of 20 wt.% lignin-based foams and control foam. Green bars indicate the foams that meet the standard requirement, and the orange bar represents the control foam without lignin.

The tear strengths of the lignin-based foams were better than the control foam made without lignin (Figure 4). The standard requirement for the automotive application, especially for the panel insulator and floor carpet, is in the range of 200–1000 N/m. All the lignin-based foams, as well as control foam, had higher tear strength values than what is required by the standard [40].

### 2.5. Compression Set

**Figure 5:**
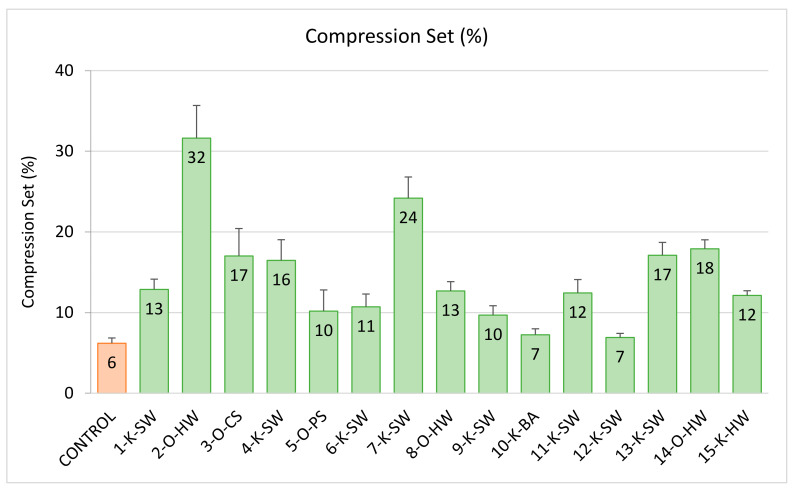
Compression set (ASTM D 3574) of 20 wt.% lignin-based foams and control (without lignin); a lower value is better. Green bars indicate the foams that meet the standard requirement, and the orange bar represents the control foam without lignin.

The compression set analysis results of flexible PU foams are shown in Figure 5. It was found that partially substituting the polyol with unmodified lignin reduced foam resiliency, resulting in a higher compression set. The lignin-containing PU flexible foams had higher compression sets (7–32%) than the control foam (6%) without lignin. This increase in the compression set is also due to an increase in crosslinking density (higher urethane linkages) as a result of the higher OH content of lignins, which was also reported previously [42]. Therefore, when the foams were kept at an elevated temperature during the compression set test (60 °C), the hydrogen bonds between N-H from urethane (i.e., hard segment) and O-H groups from polyol/lignin were weakened, leading to the deformation of flexible PU foams [42]. This deformation was more evident in lignin-based foams compared to the control foams. However, all the lignin-based foams had the compression set values well within the required standard limit (below 60%) for automotive applications (especially for the panel insulator or floor carpet) [40].

### 2.6. Tensile Strength and Ultimate Elongation

The tensile strengths of all the lignin-based foams were either in the same range as of control or higher than control, as shown in Table 2. The control formulation had a tensile strength of 64 kPa, and the lignin-based foams had a tensile strength in the range of 51–84 kPa. The standard range of tensile strengths for automotive applications (panel insulator or floor carpet) is in the range of 50–200 kPa [40,41]. All the lignin-based foams with a 20% replacement surpassed the minimum required value of 50 kPa, as seen in Table 2. The increase in tensile strength is due to the aromatic structure of lignin and its higher hydroxyl content, which results in a higher degree of crosslinking [27,33].

The ultimate elongation data (Table 2) shows that lignin addition decreases the ultimate elongation values of the foam compared to the control formulation (123 ± 5%). This decrease in the ultimate elongation values is potentially due to the fact that the polyol used in the control formulation has a long aliphatic chain, which gives the foam higher flexibility and elasticity. In contrast, lignin has aromatic structures [2]. As expected, the partial replacement of the soft segment of the foam (polyol portion) with an aromatic biopolymer (lignin), which has high T_g_ values (84–166 °C), reduced foam flexibility.

### 2.7. Support (Sag) Factor

The support factor assesses the foam’s ability to support the weight. All the lignin-based foams had higher support (sag) factor than control foam (without lignin), indicating that replacing even 20 wt.% of the polyol with lignins can significantly improve the support factor of PU flexible foams, as shown in Table 2.

### 2.8. Thermal Properties

Thermogravimetric analysis (TGA) was used to study the thermal degradation of PU foams. To evaluate the thermal stability of the lignin-based foams, the temperature of onset (T_onset_), the temperature of offset (T_offset_), and temperature at maximum degradation (T_max_) were recorded using TGA (Table 3.).

It was found that after partially substituting the polyol with unmodified lignin, T_onset_, T_offset_, and T_max_ of lignin-containing PU, flexible foams increased compared to the control foam. T_max_ for the control foam was 374 °C, while lignin-substituted foam had T_max_ in the range of 377–395 °C. Based on the acquired data, it was evident that lignin-based flexible PU foams had higher thermal stability than the control foam without lignin, as shown in Table 3. 

**Figure 6:**
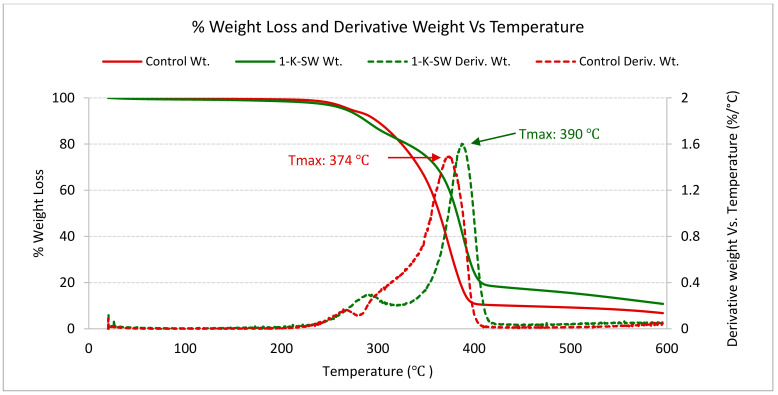
TGA and Derivative TGA curves of the produced PU flexible foams. All thermograms were acquired at a constant heating ramp of 10 °C/min in a nitrogen atmosphere.

TGA is also a very useful technique for studying the degradation steps of PU materials. Figure 6. displays the TGA curves and the derivative curves of the studied control and lignin-based (1-K-SW) PU flexible foams, respectively. Javni et al. [43] studied the thermal degradation stages of a biobased (vegetable oil-based) polyurethane network with TGA and reported that the polyurethane showed multiple (two or three) degradation. The derivative (Figure 6) presented two degradation phases for the control foam as well as (1-K-SW) lignin-based foam, indicating a similar degradation mechanism. In the control formulation without lignin, the first stage of thermal degradation started around 260 °C. While, in the lignin-based (1-K-SW) formulation, thermal degradation for the first stage started at a higher temperature, about 290 °C. The first degradation phase in the lignin-based foam can be associated with the irreversible cleavage of urethane bonds, which happens around 200 °C [4]. The second degradation phases at around 350 °C and 400 °C can be associated with the thermal degradation of soft segments polyol and lignin, as reported in the literature [44].

### 2.9. SEM Analysis

**Figure 7:**
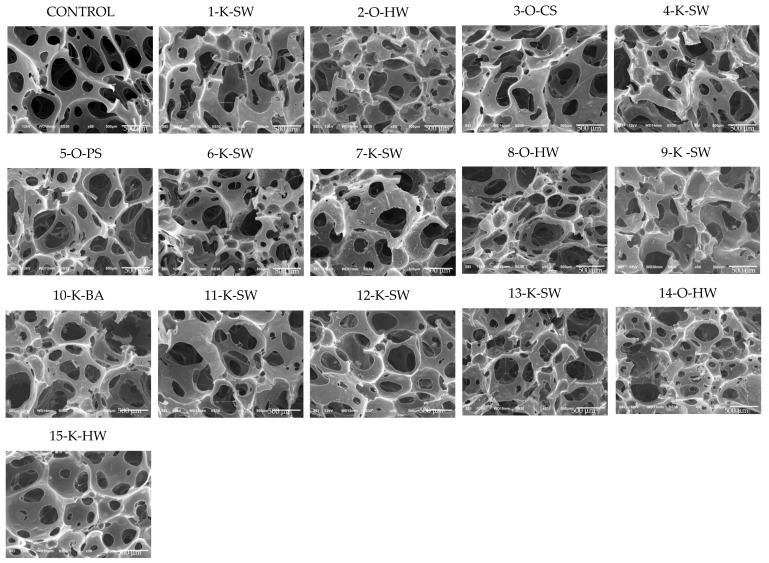
SEM micrographs (50×) of control and 15 different lignin-based flexible PU foams. K = Kraft, O = Organosolv; SW = Softwood, HW = Hardwood, BA = Bagasse, PS = Peanut Shell, CS = Corn Stover. Numbers 1–15 represent different lignin samples.

The morphologies of developed foams were assessed using scanning electron micrographs (SEM). This characteristic is very important as the mechanical performance of the foam is highly influenced by the thickness of cell walls and the average cell size [45]. Figure 7 shows SEM micrographs of control foam as well as foams made using 15 different lignins as partial polyol replacement. As expected, all the characteristic formulations had open-cell structures. SEM micrographs showed that the lignin-based foams produced smaller cell sizes and thicker cell walls (struts) than the control formulation, explaining the higher density values and mechanical properties.

### 2.10. Statistical Analysis (Pearson Correlation)

SAS software was used to analyze the correlation between lignin properties (Table 1) and measured foam performances. Among the tested properties, a positive correlation was observed between density and the total hydroxyl (OH) content of lignin (Table 4). Similarly, CFD was positively correlated with the total hydroxyl (OH) content of lignin. This is because lignins with higher hydroxyl content tend to form a more rigid structure with higher crosslinking density, which indirectly means forming thicker struts (cell walls), and hence higher density and higher CFD foams. In contrast, higher crosslinking will reduce foam flexibility and decrease the ultimate elongation of the foams.

## 3. Materials and Methods

### 3.1. Materials

For the synthesis of flexible polyurethane foam, all the foam raw materials were kindly provided by Huntsman Corporation. Including polyether-based polyol with OH number–28 mg KOH/g (OH content of 0.50 mmol/g), amine-based blowing catalyst, polymerization catalyst, and a mixture of methylene diphenyl diisocyanate (MDI) and polymeric diphenylmethane diisocyanate (pMDI) with an isocyanate content of 28% (%NCO), as reported in the product technical datasheet. Distilled water was used as the chemical blowing agent. Momentive Performance Materials Inc. provided a silicone-based surfactant. All the chemicals were used as received. Commercial lignin samples shown in (Table 1) were purchased from or supplied by different lignin producers and were used without any further chemical modification. All lignin samples were sieved using mesh No. 80 (180 μm) and then oven-dried at 105 °C for 1 h before using in the polyurethane foam formulations.

### 3.2. Lignin Characterization

#### 3.2.1. Hydroxyl Content (mmol/g)

Quantitative phosphorous nuclear magnetic resonance (^31^P NMR) spectra were recorded on Agilent DDR2 500 MHz NMR spectrometer with 7600AS 96 sample auto-samplers, running VnmrJ 3.2A with a relaxation delay of 5s, pulse angle of 90°, and with 256 scans. The solvents were composed of 300 μL DMF and 325 μL mixture of (anhydrous pyridine/deuterated chloroform) in the ratio (1.6:1, *v/v*). Forty milligrams of oven-dried lignin sample was well dissolved in the mixture of the aforementioned solvents. Then, 100 μL of cyclohexanol (22.01 mg/mL) was added as an internal standard. Followed by 50 μL of chromium (III), acetylacetonate solution (5.6 mg/mL) was used as relaxation reagent and 100 μL of 2-chloro-4,4,5,5-tetramethyl-1,3,2-dioxaphospholane as phosphitylation reagent. Spectra were processed using MestreNova software (Mestrelab Research, Version 12.0.3). A more detailed analysis results are shown in the Supplementary Materials. Quantitative ^31^P NMR is one of the most used techniques for analysis of different hydroxyl functional groups of lignin (mmol/g) [46].

#### 3.2.2. Molecular Weight Distribution

A gel permeation chromatography system (Waters company with THF as mobile phase) was used for molecular weight measurements. Due to the inadequate solubility of some lignin samples in THF (tetrahydrofuran) solvent, lignin samples were first fully acetylated using a solution of pyridine and acetic anhydride using the following procedure. First, 1 g of lignin was added to 40 mL of pyridine-acetic anhydride solution (50–50 *v/v*%) [47]. The solution was then mixed for 24 h (600 rpm) at room temperature (24 ± 2 °C). Then, to precipitate the acetylated particles, 150 mL of hydrochloric acid (HCl) with a pH of 1 was added to the solution. Precipitates were filtered using a vacuum, and the residual solids were washed with hydrochloric acid (0.05 M) solution three times, followed by deionized water several times. Acetylated lignin was dried using a vacuum oven at 40 °C for 16 h [48]. Then, THF (HPLC) grade was used to dissolve samples with a concentration of 5 mg/mL, and a syringe filter (PTFE, 0.45 μm) was used to filter. The filtrate was used for GPC analysis. A GPC system was then used to examine the filtrate flow at 1 mL/min rate, using the following columns: 300 mm × 7.8 mm Waters columns in series including 1-Styragel HR 4 THF (5k–600k Å), 2-Styragel HR 3 THF (500–30k Å), and 3-Ultrastyragel THF (100–10k Å). Polystyrene standards of molecular weights (162, 370, 580, 945, 1440, 1920, 3090, 4730, 6320, 9590, 10,400, and 16,200 Da) were used in calibration. Twenty-five microliters filtrate solution was injected into the system, and a RI detector was used, which was constantly kept at 35 °C temperature during the analysis. Data were collected and analyzed using Empower GPC Software.

#### 3.2.3. Glass Transition Temperature (T_g_)

A differential scanning calorimeter (DSC 6000, PerkinElmer) was used to examine the glass transition temperature (T_g_) of all the lignin samples. Approximately 10 mg of lignin was weighed in a sealed, hermetic aluminum pan, and first heated from room temperature (23 ± 2 °C) to 120 °C; at a heating rate of 20 °C/min for its first heating cycle and then cooled at a cooling rate of 20 °C/min to 25 °C and then kept isothermal for 10 min. The second heating cycle was applied at a heating rate of 20 °C/min to 200 °C. All the cycles were run in the N_2_ environment. The second heating cycle was used to determine the glass transition temperature (T_g_) of lignin samples.

### 3.3. Synthesis of Lignin-Based Flexible Polyurethane Foams

Polyol was added in a 12 oz cup followed by the sequential addition of water, gelation catalyst, blowing catalyst, and silicone-based surfactant in a certain proportion indicated in Table 5. Then solid lignin powder (Sieved 80 mesh and oven-dried) was added (amount equivalent to 20 wt.% of total polyol), and the mixture was mixed thoroughly for 2 min at 2000 rpm using a high-speed digital overhead stirrer to ensure homogenous mixing. Then, isocyanate was added to the polyol component, and the solution was mixed vigorously at 2000 rpm for 4–5 s. After that, the mixture was immediately poured into the silicone molds to rise in free expansion at 60 °C for an hour and then air-dried at room temperature for 24 h to ensure complete curing before characterization.

Fifteen lignins were employed using the same formulation where 20 wt.% of the total polyol used in control foam formulation was replaced with lignin, while keeping the isocyanate amount constant in all the formulations, including the control. The isocyanate index (NCO index) for the control formulation was 80. For the lignin-based foams, only the polyol content was changed to 80 PPHP, and lignin of 20 PPHP was added while keeping all the other materials constant (the same as control, shown in (Table 5). As the lignin’s equivalent weight was lower than polyol, after replacing 20 wt.% of the polyol with lignin and keeping the isocyanate amount the same as control, the isocyanate index for all lignin-based polyurethane foams decreased and were in the range of 60–75 (NCO index). The main reason for not changing the amount of NCO addition was to see the sole effect of different lignins. Isocyanate amount greatly impacts foam performances. Changing the NCO content based on calculated lignin’s OH content would have been only helpful in this comparative study if all of the OH groups of lignin were reacting with NCO. The other main challenge is the significant difference between the reactivity of aliphatic OH groups of lignin with the reactivity of different phenolic OH groups of lignin (guaiacyl, springy, and hydroxyphenyl) with NCO. Using stoichiometric NCO addition would mask the effect of all other lignin properties on foam performance.

### 3.4. Characterization of Lignin-Based Flexible PU Foam

#### 3.4.1. Apparent Density

The apparent density of the foam was determined following ASTM D3574–17 standard test method. The specimen size was 50 mm × 50 mm × 25 mm (Length × Width × Height), and the apparent density was calculated by taking the ratio between weight (M) and volume (V) of the specimens and reported in kg/m^3^.

#### 3.4.2. Tensile Strength and Ultimate Elongation

The tensile test was performed based on the ASTM D3574–17 standards with rectangular-shaped specimens. Instron 5565 Universal Testing Machine was used to determine the tensile strength (kPa). The test was performed with a minimum grip separation of 62.5 mm at a grip crosshead separation speed of 500 ± 50 mm/min. Ultimate Elongation was measure using Equation (1).

(1) $$\%\;\mathrm{Ultimate}\;\mathrm{Elongation}=\frac{\left[d_{f}-d_{o}\right]}{d_{o}}$$

where:

d_o_ = original distance between grips (which was set to 62.5 mm in all the tests)

d_f_ = distance between the grips at rupture/breakpoint.

#### 3.4.3. Tear Strength

**Figure 8:**
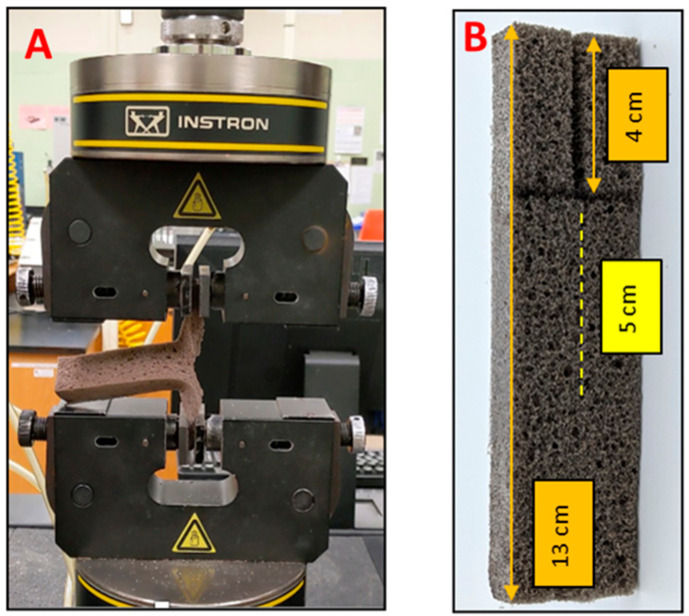
(**A**)Testing tear strength on the lignin-based foam using Instron 5565 Universal Testing Machine (**left**); (**B**)Tear Test Specimen (**right**).

The tear test was performed on the Instron Universal 5565 testing Machine (shown in Figure 8) following the ASTM D3574 standard test method. Figure 8B shows the specimen for the tear test with a rectangular-shaped slit. The specimen is clamped in the jaws of the Instron 5565 Universal testing machine, ensuring that the jaws grip the specimen uniformly and properly, as shown in Figure 8A. The crosshead speed of grip separation was 500 ± 50 mm/min. Tear strength was calculated using maximum force registered on the Instron 5565 Universal testing Machine and the average thickness of the specimen as given by Equation (2).

(2) $$\mathrm{Tear}\;\mathrm{strength}\;\left(\frac{N}{m}\right)=\frac{F}{T}\times 10^{3}$$

where:

F = force, N, and

T = thickness, mm

At least three replicates per formulation were tested, and the average values were reported in N/m.

#### 3.4.4. Compressive Force Deflection (CFD, at 50 % Compression)

**Figure 9:**
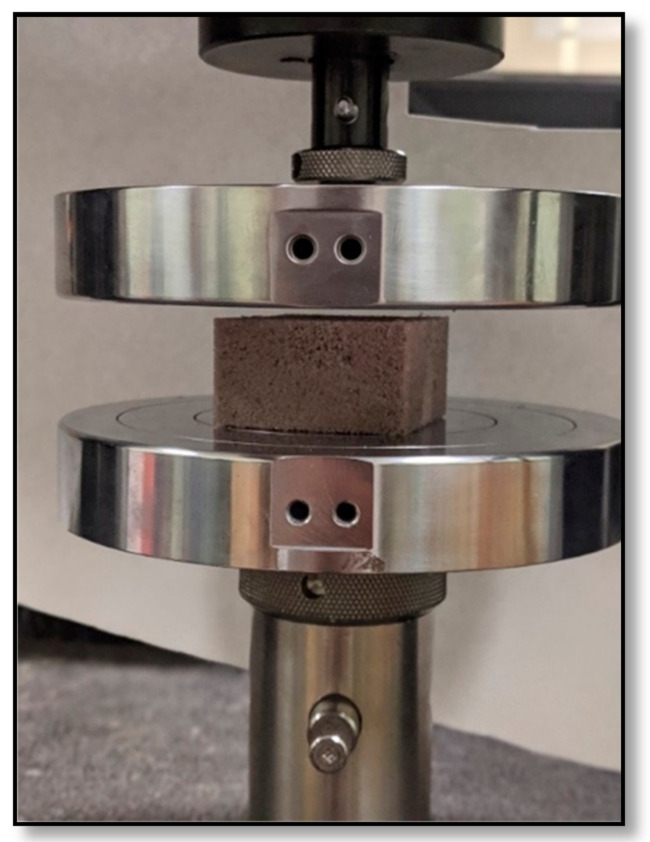
Compression force deflection (CFD) test on lignin-based flexible PU foam using Instron 5565 Universal testing machine.

Compressive force-deflection (CFD) measures the force needed to produce a 50% compression over the foam specimen’s entire upper area. For this test, the Instron 5565 Universal testing machine (shown in Figure 9) was used, and ASTM D 3574 -17 test method was followed. The samples were compressed twice, first to a deflection of 75% and then to 80% of the original thickness. The specimen was compressed at a rate of 50 ± 5 mm/min to 50% of its original thickness, and the final force was recorded, in N, after 60 ± 3 s, while keeping the specimen compressed. The compression stress at a 5–6% compression strain was used to calculate the compression modulus of the samples.

#### 3.4.5. Support (Sag) Factor

The support factor (the ratio of 65% CFD and 25% CFD values) was calculated to evaluate the cushioning performances of foams using the Instron 5565 Universal testing machine. CFD at 65% compression and 25% compression was calculated following the ASTM D 3574-17 test method. The following equation was used to calculate the support factor Equation (3).

(3) $$\mathrm{Support}\;\mathrm{factor}=\frac{\mathrm{CFD}\;65\%}{\mathrm{CFD}\;25\%\;}$$

#### 3.4.6. Compression Set

This test method comprises compressing the foam sample to a specified deflection at a specific temperature and duration, then evaluating the change in the specimen’s height after a certain recovery period. The compression device was comprised of two flat, stiff metal plates arranged in such a manner that the plates were facing parallel. The space between the plates was modifiable to the required compression using spacers and clamps. All measurements, conditioning, and recovery of the test specimens were conducted at room temperature (23 ± 2 °C) following ASTM D3574–17 standard. The test specimens (dimensions of 50 mm length × 50 mm width × 25 mm height) were placed in the compression device, and the plates were deflected to 6-mm thickness using a spacer and the clamps. The compression set up containing a deflected specimen was placed in an oven at 60 ± 2 °C for 22 h. After that, the compression device was taken out of the oven, and the samples were removed immediately, allowing them to recover for 30 to 40 min. The final thickness of the test specimen was measured precisely using the Vernier calipers, and the following equation Equation (4) was used to calculate the compression set values.

(4) $$C_{d}=\left[\frac{t_{o}-t_{f}}{t_{o}-t_{s}}\right]\times 100$$

where:

C_d_: compression set expressed as a percent of the original deflection,

t_o_: the original thickness of the test specimen,

t_s_: the thickness of spacer bar used, and

t_f_: the final thickness of the test specimen.

At least three replicates per sample were tested, and their average and the standard deviation were reported.

#### 3.4.7. Thermal Properties (TGA)

A thermogravimetric analyzer (TGA Q50) from TA instruments was used to evaluate the thermal stability of the lignin-containing PU flexible foams. Approximately 10 mg specimen was cut from the core of the foam. The specimen was heated from room temperature to 600 °C at a rate of 10 °C/min in a nitrogen atmosphere. The temperature of onset, the temperature of offset, and temperature at maximum weight loss were reported.

#### 3.4.8. SEM

Scanning electronic microscopy (SEM) was performed with a JEOL 6610LV scanning microscope to study and evaluate the cellular structure of the foam. The samples were coated with gold nanoparticles using a vacuum gold sputter coater (EMscope SC500) before SEM analysis. The accelerating voltage of 12 kV, spot size 30, and a magnification of 50× were used.

#### 3.4.9. Statistical Analysis

The results were analyzed using SAS University Edition software to check the analysis of variance (ANOVA) and Tukey’s honestly significant difference (Tukey’s HSD) test with a 95% confidence level to calculate the significant differences between the means. The correlations between lignin properties and lignin-based flexible foam performance were also analyzed using the Pearson correlation matrix in SAS University Edition software.

## 4. Conclusions

In this study, lignin-based flexible polyurethane foams were synthesized by partially substituting (20 wt.%) the petroleum-based polyols with 15 different unmodified lignins (without any pretreatment or modification). Lignin incorporation improved the foams’ mechanical properties, such as compression force deflection (CFD), compression modulus, tear resistance, and support factor. Also, lignin addition increased the tensile strength of the foam while it decreased the ultimate elongation. All the lignin-based foams had higher thermal stability than control foam (without lignin). The Pearson correlation analysis of lignin properties with the performance of lignin-based PU flexible foams showed that total hydroxyl content was the most influential lignin properties affecting the performance of lignin-based flexible PU foams. It was seen that lignins with higher OH content resulted in foams with higher density and compression force deflection. Overall, among tested lignins, organosolv lignins (in solid form) seemed to be more suitable lignins for partially replacing fossil fuel-based polyol in the formulation of PU flexible foams intended for automotive applications. This might be due to the fact that organosolv lignins had higher solubility in the co-polyol (used for foam formulation), higher flexibility (lower glass transition temperature, T_g_)_,_ and most importantly, lower hydroxyl content. Kraft lignins yielded higher density foams with better mechanical properties than control foam, making them possibly more suitable for heavy-duty foam formulations. The future work will be focused on using the most suitable lignins identified in this study and formulate foams with higher lignin content (30–50 wt.%) while ensuring that developed foams still meet the OEM performance requirements.

## Figures and Tables

**Table 1:** List of lignin samples used in the flexible PU foam formulations and their measured properties.

No.	Process	Source	ID	Hydroxyl Content ^a^ (mmol/g)	Mn (Da)	Mw (Da)	PDI	T_g_ (°C)
1	Kraft	Softwood	1-K-SW	5.61	1990	6580	3.31	149
2	Organosolv	Hardwood	2-O-HW	4.56	1280	3440	2.69	104
3	Organosolv	Corn Stover	3-O-CS	3.90	1750	6240	3.57	143
4	Kraft	Softwood	4-K-SW	5.92	1770	6070	3.43	148
5	Organosolv	Peanut Shell	5-O-PS	3.48	1640	9080	5.54	96
6	Kraft	Softwood	6-K-SW	5.72	2030	8090	3.99	166
7	Kraft	Softwood	7-K-SW	5.37	1750	5510	3.15	155
8	Organosolv	Hardwood	8-O-HW	4.08	1640	4070	2.48	134
9	Kraft	Softwood	9-K-SW	6.70	2080	6920	3.33	152
10	Kraft	Bagasse	10-K-BA	4.63	2000	6550	3.28	147
11	Kraft	Softwood	11-K-SW	6.42	1540	4290	2.79	148
12	Kraft	Softwood	12-K-SW	5.22	2250	12100	5.38	158
13	Kraft	Softwood	13-K-SW	3.49	2175	7280	3.35	138
14	Organosolv	Hardwood	14-O-HW	5.19	1490	4250	2.85	84
15	Kraft	Hardwood	15-K-HW	6.79	1030	1740	1.69	134

^a^: Hydroxyl content is based on the total OH content of lignin (Aliphatic OH + Ph-OH and –COOH).

**Table 2:** Tensile Strength, Compression Modulus, Sag Factor, and Ultimate Elongation of 20% lignin-based foams and control (without lignin), (an average of at least three replicates ± standard deviation).

Foam Samples	Tensile Strength (kPa)	Compression Modulus (kPa)	Sag Factor	Ultimate Elongation (%)
CONTROL	64 ± 6	18 ± 2	3.0 ± 0.2	123 ± 5
1-K-SW	62± 7	36 ± 5	5.1 ± 0.5	61 ± 4
2-O-HW	63 ± 5	18 ± 2	6.8 ± 1.1	63 ± 7
3-O-CS	74 ± 8	31 ± 5	3.7 ± 0.1	75 ± 7
4-K-SW	59 ± 1	45 ± 7	5.0 ± 0.3	53 ± 6
5-O-PS	72 ± 1	24 ± 3	3.9 ± 0.2	77 ± 1
6-K-SW	62 ± 2	26 ± 3	6.4 ± 0.6	67 ± 2
7-K-SW	61 ± 9	29 ± 3	4.9 ± 0.3	61 ± 7
8-O-HW	62 ± 6	19 ± 3	14.1 ± 1	75 ± 9
9-K-SW	51 ± 5	29 ± 4	5.7 ± 0.8	37 ± 5
10-K-BA	69 ± 5	31 ± 3	3.9 ± 0.3	65 ± 4
11-K-SW	66 ± 5	37 ± 3	4.9 ± 0.3	52 ± 1
12-K-SW	74 ± 2	41 ± 6	3.6 ± 0.2	68 ± 3
13-K-SW	55 ± 3	18 ± 3	6.0 ± 0.5	68 ± 3
14-O-HW	57 ± 4	18 ± 2	3.6 ± 0.1	83 ± 12
15-K-HW	84 ± 6	55 ± 9	3.9 ± 0.3	72 ± 3

**Table 3:** T_onset_ and T_offset_ data for the TGA curves of 20% lignin-based foams and control (without lignin).

Lignin	T_onset_ (°C)	T_offset_ (°C)	T_max_ (°C)
Control	331 ± 1	390 ± 1	374
1-K-SW	356 ± 5	403 ± 5	390
2-O-HW	354 ± 1	406 ± 2	391
3-O-CS	345 ± 4	400 ± 5	386
4-K-SW	352 ± 4	399 ± 4	387
5-O-PS	344 ± 2	401 ± 2	387
6-K-SW	355 ± 2	407 ± 2	387
7-K-SW	353 ± 9	402 ± 8	390
8-O-HW	353 ± 3	403 ± 2	389
9-K-SW	345 ± 2	392 ± 3	382
10-K-BA	331 ± 13	396 ± 5	377
11-K-SW	352 ± 2	400 ± 3	388
12-K-SW	363 ± 2	412 ± 6	395
13-K-SW	358 ± 2	408 ± 1	381
14-O-HW	342 ± 3	392 ± 2	392
15-K-HW	346 ± 2	394 ± 2	381

Note: SW = Softwood, HW = Hardwood, PS = Peanut Shell, CS = Corn Stover, K = Kraft, O = Organosolv, BA = Bagasse.

**Table 4:** Summary of Pearson Correlation Matrix.

Pearson Correlation Coefficient	Total OH Content of Lignin	*p*-Value
Density	0.68	<0.0001
CFD	0.69	<0.0001

**Table 5:** Formulation in Parts Per Hundred Polyol (PPHP) and grams for the synthesis of lignin-based PU foams.

Components	PPHP *	Actual Weight (g)	PPHP *	Actual Weight (g)
	Lignin-Based	Lignin-Based	Control	Control
Copolyol (Polyether polyol)	80	20	100	25
Lignin	20	5	−	−
Water	2.5	0.63	2.5	0.63
Gelation catalyst	0.53	0.13	0.53	0.13
Blowing catalyst	0.32	0.08	0.32	0.08
Surfactant	0.8	0.20	0.8	0.20
Diisocyanate	−	10.05	−	10.05

* Parts Per Hundred Polyol.

## Data Availability

Not Applicable.

## Supplementary Materials

The following data are available online. A more detailed ^31^P NMR analysis results of lignin samples and correlation analyses are shown in Table S2: List of lignin samples used in the flexible PU foam formulations and their measured OH content (^31^P NMR results), Table S3: Summary of Pearson Correlation Matrix of Density and CFD with Aliphatic OH, Table S4:Summary of Pearson Correlation Matrix of Density and CFD with Phenolic OH, Table S5: Summary of Pearson Correlation Matrix of Density and CFD with Carboxylic OH in the supplemental information file.

## Sample Availability

Samples of the compounds are not available.
