# Supplementary material for: Lignin as a Partial Polyol Replacement in Polyurethane Flexible Foam
Source: Molecules. 2021 Apr 15;26(8):2302. doi: 10.3390/molecules26082302 (PMC8071504; doi:10.3390/molecules26082302)
Supplement: Supplementary file 1 [file molecules-26-02302-s001.zip › molecules-1154756-supplementary.pdf]

Supplementary Information

Table S1: List of lignin samples used in the flexible PU foam formulations and their measured OH content (^{31}P NMR results).

No.	Process	Source	ID	Aliphatic-OH (mmol/g)	Ph-OH (mmol/g)	COOH (mmol/g)	Total Hydroxyl Content (mmol/g)
1	Kraft	Softwood	1-K-SW	2.0	3.20	0.43	5.61
2	Organosolv	Hardwood	2-O-HW	1.4	2.83	0.32	4.56
3	Organosolv	Corn Stover	3-O-CS	2.3	1.11	0.53	3.90
4	Kraft	Softwood	4-K-SW	2.0	3.41	0.48	5.92
5	Organosolv	Peanut Shell	5-O-PS	1.3	1.87	0.26	3.48
6	Kraft	Softwood	6-K-SW	1.9	3.42	0.41	5.72
7	Kraft	Softwood	7-K-SW	1.8	3.15	0.43	5.37
8	Organosolv	Hardwood	8-O-HW	0.8	3.03	0.26	4.08
9	Kraft	Softwood	9-K-SW	1.8	4.39	0.56	6.70
10	Kraft	Bagasse	10-K-BA	3.6	0.49	0.54	4.63
11	Kraft	Softwood	11-K-SW	2.4	3.71	0.37	6.42
12	Kraft	Softwood	12-K-SW	1.8	2.86	0.58	5.22
13	Kraft	Softwood	13-K-SW	1.5	1.92	0.04	3.49
14	Organosolv	Hardwood	14-O-HW	1.7	3.29	0.24	5.19
15	Kraft	Hardwood	15-K-HW	1.2	5.44	0.16	6.79

Correlations between the Density and CFD with the Aliphatic, Phenolic, and Carboxylic OH content of lignins are shown in Table 2-4. As can be seen, most of them are not statistically significant. Only there is a significant correlation between CFD and phenolic OH of lignin (p-value of 0.036), but the correlation is very low (0.54).

Table S2: Summary of Pearson Correlation Matrix of Density and CFD with Aliphatic OH

Pearson Correlation Coefficient	Aliphatic OH	*P*-value
Density	0.0079	0.977
CFD	0.0951	0.735

Table S3:Summary of Pearson Correlation Matrix of Density and CFD with Phenolic OH

Pearson Correlation Coefficient	Phenolic OH	*P*-value
Density	0.487	0.065
CFD	0.542	0.036

Table S4: Summary of Pearson Correlation Matrix of Density and CFD with Carboxylic OH

Pearson Correlation Coefficient	Carboxylic OH	*P*-value
Density	0.0574	0.8387
CFD	0.2804	0.3114
